# Research on the manifestations and key factors of digital competence of China’s preschool teachers

**DOI:** 10.3389/fpsyg.2026.1888962

**Published:** 2026-09-03

**Authors:** Jie Meng, Yu Ouyang, Chenggang Wang

**Affiliations:** 1 Faculty of Public Administration, Wuhu Vocational Technical University, Wuhu, China; 2 The Affiliated Kindergarten of Anhui Normal University, Anhui Normal University, Wuhu, China; 3 School of Educational Science, Anhui Normal University, Wuhu, China

**Keywords:** digital competence, internal motivation, preschool teacher, proactive personality traits, self-efficacy

## Abstract

Grounded in an integrated framework of the Iceberg Theory, TPACK, and DigCompEdu, this study developed a five-dimensional model and employed mixed methods to investigate the current status and key determinants of digital competence among Chinese preschool teachers. A quantitative survey was administered to 599 practitioners, followed by semi-structured interviews with 15 teachers. The results demonstrated that: (1) Overall digital literacy was at a moderate-to-high level. Among the five dimensions, “Digital Management Competence” scored the highest (*M* = 4.323), whereas “Digital Leadership” scored the lowest (*M* = 4.021). Scores for “Digital Education Competence,” “Digital Learning Competence,” and “Basic Digital Skills” fell within the moderate range *(M* = 4.062–4.186). (2) Demographic variables significantly influenced digital literacy. Female teachers exhibited significantly higher digital literacy than their male counterparts [*t*(140.19) = −2.61, *p* < 0.01, η^2^ = 0.012]. ANOVA revealed significant differences across educational backgrounds; notably, teachers with master’s degrees and those with bachelor’s degrees or below scored significantly higher than doctoral degree holders [*F*(2, 596) = 37.371, *p* < 0.001, η^2^ = 0.111]. Teachers with over 11 years of experience demonstrated the highest literacy levels (*F* = 4.286, *p* = 0.001). Regarding professional titles, senior teachers scored significantly higher than junior or ungraded teachers, while intermediate title holders exhibited a distinct “low point” [*F*(3, 595) = 12.717, *p* < 0.001, η^2^ = 0.060]. (3) Regression analysis identified self-efficacy, intrinsic motivation, and proactive personality traits as the most significant predictors of digital literacy. (4) Qualitative findings highlighted that while educators recognize the value of digital competence, they face systemic challenges such as inadequate training and uneven resource distribution. Targeted institutional support is recommended to enhance teachers’ self-efficacy and motivation, thereby fostering their digital development.

## 1 Introduction

Basic information technology (IT) skills, such as retrieving, evaluating, storing, and generating information, are fundamental for preschool teachers adapting to the digital environment with digital tools not only boosts teachers’ confidence and positive attitudes but also encourages them to integrate these tools into their pedagogical practices (Forsling, 2023; Li et al., 2024). By leveraging interactive whiteboards, educational applications, and intelligent content creation (e.g., designing slides or sourcing relevant images), teachers can transform thematic activities and extend course content (Leh, 2022). The integration enhances work efficiency while making learning activities more vivid and engaging, ultimately leading to better learning outcomes. Moreover, strong digital competence enables teachers to design innovative, practice-based activities that stimulate children’s curiosity and capacity for autonomous learning. Beyond the classroom, the judicious use of digital technology can also help bridge the digital divide caused by geographical and socioeconomic disparities, thereby ensuring equitable access to digital resources for all children (Zhou et al., 2025).

Despite the recognized importance of digital competence, the majority of existing research has focused on teachers in primary, secondary, and higher education (Zheng, 2019; Yang et al., 2024), with very few studies specifically investigating preschool teachers (Herman et al., 2025). This gap is critical because the digital competence required in early childhood education (ECE) differs fundamentally from that in other educational stages (Valdez and Mendoza, 2024). Unlike secondary or higher education, which often emphasize the systematic transmission of professional knowledge, ECE prioritizes integrating digital tools into play-based, interactive activities to nurture children’s initial perceptions of technology. Preschool teachers must bridge the digital and physical worlds, using virtual simulations to help young children better understand and perceive reality (Herman et al., 2025). Thus, enhancing their digital competence is essential for boosting teaching creativity, optimizing gamified learning environments, and supporting children’s language, cognitive, and social development (Gwo et al., 2020; Forsling, 2023).

Yet, two significant gaps remain in the current literature. First, most studies examine influencing factors from a single perspective, overlooking the interdependence of variables in the highly interactive ECE environment (Enochsson and Ribaeus, 2021). As a result, there is still no consensus on the primary predictors of preschool teachers’ digital competence. Second, empirical evidence regarding the status and determinants of digital competence among Chinese preschool teachers remains scarce. This is particularly pressing given that, in 2025, the Chinese government released The Chinese Smart Education White Paper Advocates for Digital Education Advancement, which explicitly calls for preschool teachers to possess robust digital competence, develop intelligent teaching environments, and guide young children in the appropriate use of technology (Ministry of Education of the People’s Republic of China., 2025).

This study bridges these gaps by proposing an integrated theoretical framework that synthesizes the Iceberg Model, DigCompEdu, and TPACK. We align Herman et al.’s (2025) TPACK framework with the “visible” surface of the Iceberg Model, conceptualizing Digital Basic Competence, Digital Teaching Competence, and Digital Management Competence as the observable integration of technological, pedagogical, and content knowledge. Meanwhile, the “submerged” part of the iceberg—representing the underlying drivers of continuous growth, such as intrinsic motivation and organizational leadership—is mapped to Digital Learning Competence and Digital Leadership Competence, drawing on Redecker’s (Redecker, 2017). DigCompEdu framework: Guided by this five-dimensional model, the study conducts a comprehensive analysis of preschool teachers’ digital competence in China, addressing three core questions:

(1) What is the current level of preschool teachers’ digital competence in China across the five dimensions?
(2) What factors influence the level of preschool teachers’ digital competence in China?
(3) What are the perceptions of preschool teachers in China regarding the use of digital tools in educational environments?

## 2 Literature review

Digital competence primarily evaluates teachers’ capacity to utilize digital technologies for teaching purposes (Redecker, 2017; Pinto-Santos et al., 2022; Magdalena et al., 2024). In the academic discourse, scholars generally distinguish between two related concepts: digital literacy and digital competence. Digital literacy focuses on the correct use, assessment, and handling of digital resources, tools, and content creation for lifelong learning (Peng and Zhu, 2024). In contrast, digital competence is a more comprehensive construct that encompasses digital literacy (Almås et al., 2021). Almås while emphasizing workplace adaptability, ethical standards, and the continuous acquisition of professional qualifications (Yang and Lee, 2023). It involves not only technical proficiency but also critical skills such as reflecting on technology use and making informed pedagogical decisions (Peng and Zhu, 2024).

To evaluate these competencies, various assessment tools have been developed globally, each with distinctive features and regional applicability. Prominent frameworks include DigCompEdu (Redecker, 2017), INTEF, TPACK (Herman et al., 2025), ISTE NETS-S, and TEDDICS (Magdalena et al., 2024). For instance, DigCompEdu delineates five fundamental competencies: information and data literacy, communication and collaboration, digital content creation, security awareness, and problem-solving skills, and proposes a distinction from novice to leader and ultimately to excellence (Redecker, 2017). Furthermore, recent research suggests that teachers should adopt a student-centered approach, leveraging emerging digital skills—such as those involving Generative Artificial Intelligence (GenAI)—to enhance students’ digital capabilities (Pinto-Santos et al., 2022).

Previous research on digital competence has focused primarily on primary and secondary school teachers, revealing notable differences. Studies indicate that teachers have limited abilities to integrate GenAI into teaching, with most preschool teachers relying on occasional digital tool use. Recent findings show that teachers’ digital competence is relatively high in digital social responsibility and awareness, lower in professional development, and weakest in digital technology knowledge, skills, and application (Song, 2023; Liu and Chen, 2026), highlighting the need for additional training (Trninić and Bokan, 2022). Although teachers hold positive attitudes toward digital teaching, they still show notable shortcomings in using digital technologies to solve practical educational problems (Liu and Chen, 2026). A survey using DigCompEdu as the primary assessment tool revealed that “digital resources” ranked highest among teachers’ competencies, followed by “teaching and learning” and “professional engagement,” while “assessment and feedback” scored the lowest (Park et al., 2024, 71–104).

Scholars have also examined background factors and found differences in primary school teachers’ digital competence according to sociological variables, such as gender, age, teaching experience, and professional title (Héctor et al., 2024). Regarding gender, female teachers demonstrated higher numerical ability than male teachers (Huang and Guo, 2024). In terms of age, younger teachers’ digital competence exceeded that of older teachers (Fethi and Deborah, 2020; Héctor et al., 2024). Teaching experience was negatively correlated with computer proficiency (Fethi and Deborah, 2020). Finally, teachers with different professional titles differed significantly in the dimension of digital teaching and learning (Héctor et al., 2024; Zhang et al., 2025)

Several studies have assessed the digital competence of primary and secondary school teachers. Using the “Digital Literacy Test Questions for Primary and Secondary School Teachers,” researchers found that primary school teachers outperform secondary school teachers (Song, 2023). Other studies using a self-designed questionnaire compared teachers across Chinese regions and reported that competence was highest in the developed eastern regions, followed by the western regions, with smaller disparities in the central regions (Song, 2023; Huang and Guo, 2024). Notably, a study applying DigCompEdu to both urban and rural primary and secondary teachers found that urban teachers scored significantly higher than rural teachers on all DigCompEdu factors; mean scores for professional engagement (PE), digital resources (DR), and teaching and learning (TL) were higher for urban teachers (Li et al., 2024)

Teachers’ digital competence is shaped by a diverse array of factors that vary across cultural, regional, and educational contexts, including assessment instruments, background characteristics, and situational determinants (Basantes-Andrade et al., 2022; Kim and Hong, 2024; Park et al., 2024). Despite this diversity, current research has predominantly focused on primary, secondary, and university teachers, leaving the preschool sector significantly under-explored (Basantes-Andrade et al., 2022). This neglect is concerning, as preschool teachers face unique expectations and challenges in the digital age (Herman). Given that digital competence levels vary markedly across educational stages, tailored assessments for preschool teachers are urgently needed (Basantes-Andrade et al., 2022; Li et al., 2024). Improving preschool teachers’ digital skills is not only a professional requirement but also a pedagogical necessity, as it enables the creation of engaging, technology-based activities and immersive environments that help children connect with the real world (Leh, 2022). According to Kim and Hong (2024), these capabilities are influenced by multifaceted determinants, including sociological, occupational, emotional, self-efficacy, motivational, and organizational factors. These factors align well with the theoretical structures of the TPACK framework and the Iceberg Model Building on this integration, this study aims to examine how these factors affect preschool teachers’ digital competence and to identify the core influences that can enhance their proficiency.

## 3 Methodology

### 3.1 Research design

This research is a key project of the school where I am employed. This study employs a mixed-methods approach (D’Agostino and Frost, 2025), who suggest that mixed-methods approaches allow researchers to examine the same issue from both qualitative and quantitative perspectives. This study employs a mixed-methods design to investigate the digital competence of preschool teachers in China. Specifically, qualitative data are used to complement the quantitative findings, thereby enhancing the rigor of the research. Our mixed-methods design process consists of three stages. In the first stage, quantitative data were collected through a questionnaire to understand the factors that influence teachers’ manifestations and digital competence. In the second stage, qualitative data were collected through semi-structured interviews to further explore and explain the reliability of the quantitative findings and thus strengthen the study’s robustness. In the final stage, the results of quantitative and qualitative data were integrated to provide a more comprehensive interpretation of the research problem.

### 3.2 Participation and sampling

#### 3.2.1 Questionnaires

The study participation comprised preschool teachers in China during the 2025–2027 period.

Through online surveys, we collected 661 questionnaires; after excluding invalid responses, a final sample of 599 questionnaires was obtained, with 90.6% validity. In our study, we collected preschool teachers from different regions of China. The sample represented diverse demographic and professional backgrounds, including age, gender, educational background, positional titles, organization type, professional background, and years of teaching years, as shown in Table 1.

**TABLE 1:** Characteristics of the participants in the questionnaire.

Variable	Level	Number	Percentage
Age	Under 25 years old	222	37.1%
	26–30 years	212	35.4%
	31–35 years	101	16.9%
	36–40 years	55	9.2%
	Older than 40 years	9	1.5%
Gender	Female	495	82.6%
	Male	104	17.4%
Educational background	Undergraduate degree or lower	540	90.2%
	Master	27	4.5%
	Doctor	32	5.3%
Positional titles	Newly hired or not yet graded	292	48.7%
	Junior professional title	216	36%
	Medium-grade professional title	77	13%
	Senior professional title	14	2.3%
Kindergarten type	Public Kindergarten	342	57.1%
	Private institution	257	42.9%
Professional background	Preschool education major	484	80.8%
	Not majoring in preschool education	115	19.1%
Teaching years	Under 3 years	270	45.1%
	4–7 years	255	42.5%
	8–10 years	47	7.8%
	More than 11 years	27	4.6%

#### 3.2.2 Interview

For the qualitative phase, the research design was structured around three key dimensions: representative sampling, strict ethical adherence, and a hybrid analytical strategy.

Using the snowballing method, 15 preschool teachers were purposively selected to ensure data richness and transferability. Participants were chosen based on diverse teaching experiences, educational backgrounds, and geographical distribution. This heterogeneity ensures that the findings capture a broad spectrum of perspectives regarding digital technology use (shown in Table 2 for details)

**TABLE 2:** Characteristics of the participants in the interview.

Interviewee code	Gender	Age	Professional title	Kindergarten type
P1	Female	54	Senior	Private
P2	Female	49	Senior	Public
P3	Female	38	Senior	Private
P4	Female	35	Intermediate	Public
P5	Male	42	Intermediate	Public
P6	Female	36	Senior	Public
T1	Female	35	Intermediate	Public
T2	Male	31	Intermediate	Public
T3	Female	36	Intermediate	Public
T4	Female	40	Junior	Public
T5	Female	34	Intermediate	Public
T6	Female	23	Junior	Private
T7	Female	32	Intermediate	Public
T8	Female	22	Junior	Private
T9	Female	33	Intermediate	Public

### 3.3 Study instruments

#### 3.3.1 Questionnaire

Through quantitative research, we aim to use a questionnaire to examine the manifestations of preschool teachers’ digital competence and understand the factors that influence early childhood educators’ digital competence. The questionnaire consisted of two sections. The first section collected participants’ demographic information, including gender, educational background, years of teaching experience, professional title, and kindergarten type. The second section of the questionnaire, through CFA validation, irrelevant or similar items were adjusted, ultimately resulting in five dimensions. The primary purpose of these five dimensions is to measure teachers’ digital competence across five dimensions, including (1) Digital Fundamental Competence, (2) Digital Teaching Competence, (3) Digital Learning Competence, (4) Digital Leadership Competence, and (5) Digital Management Competence^1^, which is shown in Table 3.

**TABLE 3:** Summary table of the dimension distribution of the questionnaire^1^.

Dimension	Number	Cronbach’s alpha	Exercise problems	Theoretical basis
Digital Fundamental Competence (Redecker, 2017; Zheng, 2019, Zhang, 2023)	9	0.953	I can mitigate cyber risks and adhere to ethical standards. I am familiar with the operation of common teaching equipment.	TPACK
Digital Teaching Competence (Zheng, 2019; Hu, 2023)	8	0.940	Using digital technologies to develop resources and perform personalized assessments.	TPACK
Digital Learning Competence (Shang and Gan, 2009; Zheng, 2019)	8	0.950	Applying digital technologies to educational activities mitigates cyber risks.	DigCompEdu, Iceberg
Digital Leadership Competence (Shang and Gan, 2009; Cai, 2024)	7	0.950	I am proficient in using office software to handle daily documents, and I can utilize digital platforms to efficiently manage children’s development records.	DigCompEdu, Iceberg
Digital Management Competence (Zheng, 2019; Cai, 2024; Peng and Zhu, 2024)	6	0.963	The timeliness of digital resources in the kindergarten, combined with the use of AI tools	TPACK

^1^This study adopts an ecosystem perspective to examine factors influencing digital competence from both subject and object perspectives. Measures for smart teaching concepts and experiences were adapted from Zhang’s (2023).

The smart teaching concept scale was modified for this study and comprises five items (e.g., *Do you actively explore and utilize digital platforms or apps to enhance your ability to organize kindergarten activities?*).

The smart teaching experience scale was likewise adapted and contains three items (e.g., *Do you use an app to share children’s work and facilitate home–school communication?*).

Together these scales produced an overall reliability coefficient of 0.904, indicating high reliability.

Proactive personality traits of preschool teachers were measured using the assessment tool from Shang and Gan (2009).

This scale comprises three items and yielded an overall reliability coefficient of 0.797, exceeding 0.7 and indicating good reliability (e.g., *Can the use of intelligent teaching enhance the classroom teaching atmosphere?*).

An adapted evaluation tool reported by Hu (2023) was used to assess teachers’ ability to select and apply AI resources.

This instrument comprises three items and produced an overall reliability coefficient of 0.851, indicating high reliability (e.g., *I can use different websites or search strategies to select various artificial intelligence tools to engage children in activities?*).

Based on the evaluation instrument of Shang and Gan (2009), adapted for this study and comprising three items, the overall reliability was 0.798, exceeding 0.7 and indicating good reliability (e.g., “*If I see a colleague struggling with digital technology, I am willing and able to help her*”).

The assessment tool developed by Peng and Zhu (2024) was adapted to produce three items, with an overall reliability coefficient of 0.915, indicating high reliability (e.g., “*What information technology tools and equipment are available in your kindergarten?*”).

Teachers’ intention to use AI anthropomorphism was evaluated using the tool of Thomas et al. (2025), adapted for this study.

This yielded three items with an overall reliability coefficient of 0.764, above the 0.7 threshold and demonstrating good reliability (e.g., “*When using AI tools to obtain resources, does she seem to understand what I need?*”).

Organizational support was measured using an adaptation of Cai’s (2024) instrument, comprising three items and yielding an overall reliability coefficient of 0.899, which indicates high reliability (e.g., “Does the kindergarten provide a good working environment and facilities?”).

A second adapted set from Cai’s (2024) study, also consisting of three items, produced a reliability coefficient of 0.846, likewise indicating high reliability (e.g., “*When I encounter difficulties in learning digital resources or platforms, I can seek help from relevant personnel at the institution?*”).

**FIGURE 1:**
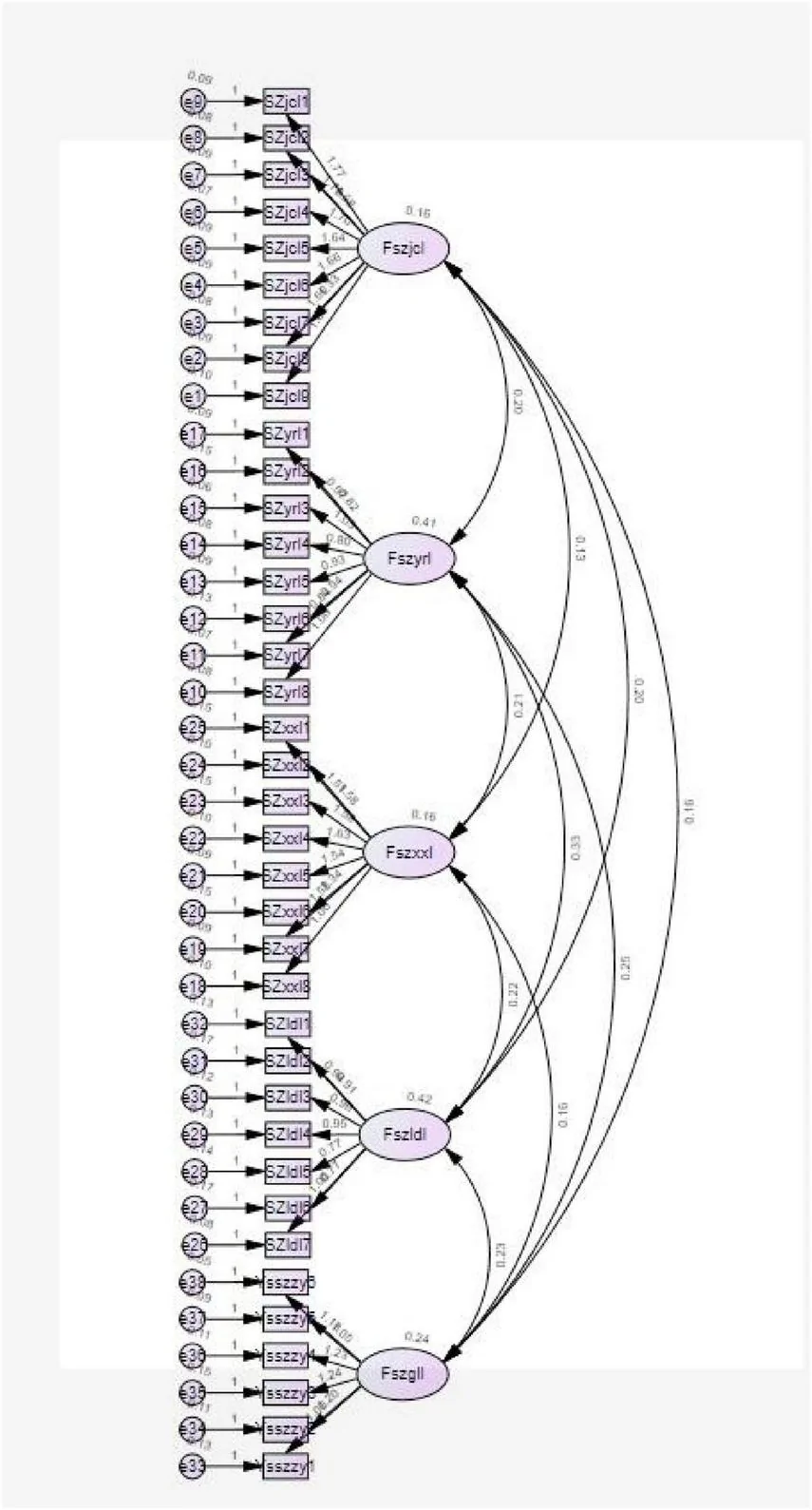
CFA illustration of model.

To ensure the reliability and validity of the instrument, we first conducted a pilot test with 100 early childhood educators. Based on the collected data, Confirmatory Factor Analysis (CFA) was performed to evaluate the structural validity of the questionnaire, specifically examining model fit indices, factor loadings, convergent validity, and discriminant validity. This section of the results is shown in Figure 1.

The results demonstrated that the questionnaire exhibited high reliability, with a Cronbach’s α coefficient of 0.925. Confirmatory Factor Analysis (CFA) was conducted using AMOS to assess structural validity. As shown in Table 4, all key model fit indices met the recommended statistical criteria. Specifically, the chi-square to degrees of freedom ratio (χ^2^/df) was 1.335 (<3.00), indicating a good model fit. The Root Mean Square Error of Approximation (RMSEA) was 0.058 (90% CI [0.048, 0.068]), which is below the critical threshold of 0.08, and the PCLOSE value was 0.100, suggesting minimal approximation error. Additionally, the Standardized Root Mean Square Residual (SRMR) was 0.018, significantly outperforming the 0.08 benchmark. For incremental fit indices, both the Comparative Fit Index (CFI = 0.952) and the Tucker-Lewis Index (TLI = 0.948) exceeded the ideal threshold of 0.90.

**TABLE 4:** Overall fit coefficient table.

Index	Reference standard	Actual measurement results
χ^2^/*df*	<3.00 is excellent	1.335
CMIN/DF	1–3 is excellence, 3–5 is good	1.335
RMSEA	<0.05 is excellence, <0.08 is good	0.058
IFI	>0.9 is excellence, >0.8 is good	0.952
TFI	>0.9 is excellence, >0.8 is good	0.948
CFI	>0.9 is excellence, >0.8 is good	0.952
NFI	>0.9 is good, >0.8 is acceptable	0.834
RFI	>0.9 is good, >0.8 is acceptable	0.821
TLI	>0.9 is excellence, >0.8 is good	0.948

Although the Goodness-of-Fit Index (GFI = 0.711) and the Adjusted Goodness-of-Fit Index (AGFI = 0.673) were slightly below the conventional cutoff of 0.90, the model is still considered acceptable given the satisfactory performance of core metrics such as RMSEA, CFI, and TLI (Byrne, 2010). In conclusion, the five-dimensional measurement model demonstrates robust structural validity.

### 3.4 Interview protocol

The interview protocol was designed to investigate in-depth data related to the research questions and to provide a deeper understanding of how digital resources and training in kindergartens, teachers’ attitudes, and current applications influence preschool teachers’ digital competence. In our interview protocol, we ask questions such as: What kinds of digital devices do you use in your teaching process? “How does digital competence affect your work efficiency?” “What specific digital support do you expect from your institution?” Do you perceive the requirements for professional title evaluation (such as digital teaching competitions or IT-based research projects) as a primary driver for improving your digital competence? Or do you feel these requirements are merely a formality? Through the interview protocol, we aimed to deepen understanding of factors influencing preschool teachers’ digital-literacy practices and support needs.

### 3.5 Data collection

Data collection was conducted in two phases. In the formal quantitative phase, the formal questionnaire was distributed to preschool teachers in China after ethical approval was obtained. To ensure consistency in questionnaire administration, participants were provided with standardized instructions before completing the survey. They were informed of the purpose of the study, the voluntary nature of participation, and the confidentiality of their responses. Participants provided informed consent before completing the questionnaire.

In the qualitative phase, we used snowballing methods to purposive select 15 preschool teachers. Assign identity codes to interviewers based on visit times, organize their conversations, and obtain interview content. To understand how they use digital technology in their teaching process, we conducted semi-structured interviews with these preschool teachers. The interviews were conducted in person and online, and lasted between 20 and 30 min. In our study, participants agreed to give permission for the interviews to be recorded. To ensure confidentiality, a rigorous pseudonymization process was implemented: real identities were replaced with alphanumeric codes (e.g., principal: P1–P6, teacher: T1–T9), and sensitive information (e.g., school, names) was masked.

### 3.6 Data analysis

Consistent with the explanatory sequential design, a hybrid coding approach combining deductive and inductive strategies was employed to enhance analytical validity:

Deductive phase: A priori codes were established based on the seven significant predictors identified in the quantitative phase (e.g., self-efficacy, organizational support). This ensured that the qualitative data directly addressed and validated the statistical model.

Inductive phase: Open coding was applied to capture emerging nuances and contextual details not covered by the survey.

This dual-process approach allowed the study to not only corroborate the quantitative results but also provide a “thick description” of the underlying mechanisms, thereby strengthening the overall explanatory power of the research.

The quantitative data were analyzed using SPSS.23. Descriptive statistics, correlations, *t*-tests, and F-tests were used to understand the baseline status, distribution differences, and correlation analysis of digital competence among preschool teachers. Regression analysis was then used to assess the predictive effects of the influencing factors and to determine how the various predictors affected digital literacy.

The qualitative data, prior to the interviews, we obtained informed consent from 15 early childhood educators, clearly informing them that participation was voluntary and that all data would be used solely for academic research purposes.

To ensure anonymity, we implemented pseudonymization during the data transcription phase. Each participant was assigned a unique alphanumeric code (e.g., teachers: T1, T2; principals: P1, P2) combined with an interview time code, while the interviewer’s name and surname were removed. The master list associating real identities with these codes is stored separately in a password-protected file accessible only to the principal investigator. Additionally, any identity-revealing information mentioned in interviews (e.g., specific school names or locations) was masked or blurred in the transcripts. All numerical data (including audio recordings and transcribed texts) are securely stored on encrypted drives to prevent unauthorized access.

Inductive and deductive approaches are used in qualitative research.

### 3.7 Ethical considerations

This study involved human participants and was conducted in accordance with the ethical requirements of Wuhu Vocational and Technical University and Anhui Normal University. The study was reviewed and approved by our institution. Participants were informed about the purpose of the study, the voluntary nature of participation, their right to withdraw, and the confidentiality of the responses. All participants signed informed consent forms before data collection. All data were securely stored on encrypted drives, accessible only to the primary researcher.

## 4 Results

### 4.1 The quantitative section

#### 4.1.1 Assessment of common method bias

Given the self-reported nature of the data, Harman’s single-factor test was employed to check for common method bias. An unrotated exploratory factor analysis extracted multiple factors with eigenvalues greater than 1. The first factor accounted for 39.18% of the total variance, which is below the 50% threshold (Podsakoff et al., 2003). This suggests that no single factor dominates the covariance structure, indicating that common method bias is not a serious concern in this study.

#### 4.1.2 The first research question

##### 4.1.2.1 Differences in digital literacy across demographic variables

The study’s first question is, “What are the factors that influence the level of preschool teachers’ digital competence in China according to the Five Digital Competence Dimensions?” To answer this question, an analysis of variance was performed using the overall dimension of preschool teachers’ digital literacy and the scores of each sub-dimension as dependent variables. The independent variables included the personal background information of preschool teachers, such as educational background, teaching experience, and professional titles (details in Table 5).

**TABLE 5:** The difference of digital literacy identity in teaching years, education background, and professional title, and sex.

Variable	Level	M ± SD	*F*	η^2^	LSD
Teaching years	Less than 3 years^1^	4.054 ± 0.031	4.286	0.021	1 < 3*; 1 < 4**; 2 < 4*
	4–7 years^2^	4.099 ± 0.515			
	8–10 years^3^	4.248 ± 0.576			
	More than 11 years^4^	4.360 ± 0.491			
Educational level	Bachelor’s degree or below^1^	4.133 ± 0.499	37.371	0.111	3 < 1***; 3***< 2
	Master’s degree^2^	4.317 ± 0.377			
	Doctoral degree^3^	3.390 ± 0.434			
Professional title	No title^1^	4.031 ± 0.523	12.717	0.060	3 < 1 < 2 < 4**
	Junior title^2^	4.231 ± 0.428			
	Intermediate title^3^	3.931 ± 0.624			
	Senior title^4^	4.517 ± 0.503			
Gender	Male teacher^1^	3.981 ± 0.055	7.11	0.012	1 < 2**
	Female teacher^2^	4.127 ± 0.023			

*P = 0.05;

**P = 0.01;

***P 0.0001.

For “Teaching years”:

^1^Less than 3 years; ^2^4–7 years; ^3^8–10 years; ^4^More than 11 years; for “Educational level”: ^1^Bachelor’s degree or below; ^2^Master’s degree; ^3^Doctoral degree; for “Professional title”: ^1^No title; ^2^Junior title; ^3^Intermediate title; ^4^Senior title; for “Gender”: ^1^Male teacher; ^2^Female teacher.

(1) Gender: Levene’s test indicated unequal variances (*F* = 7.110, *p* = 0.008); therefore, Welch’s *t*-test results were interpreted. The analysis revealed a significant difference between males (*M* = 3.98, SD = 0.055)and females (*M* = 4.13, SD = 0.023) *t*(140.19) = −2.61, *p* < 0.01, η^2^ = 0.012. Contrary to some stereotypes, female teachers demonstrated significantly higher levels of digital literacy than their male counterparts. However, the effect size was small (Cohen’s *d* ≈ 0.28).
(2) Teaching experience: The ANOVA results showed a significant but small effect, *F*(3, 595) = 4.286, *p* = 0.005,η^2^p = 0.021. *Post hoc* comparisons revealed that teachers with more than 11 years of experience (*M* = 4.36, SD = 0.491) had significantly higher digital literacy than those with 8–10 years (*p* = 0.013) and 11–15 years of experience (*p* = 0.003). No other pairwise differences were statistically significant.
(3) Educational background: The one-way ANOVA revealed significant differences in digital competence scores, *F*(2,596) = 37.371, *p* < 0.001, η^2^ = 0.111. *Post hoc* LSD tests indicated that teachers with a a master’s degree (*M* = 4.317, SD = 0.377, *p* < 0.001) reported significantly higher digital literacy identity, than those with a bachelor’s degree or below (*M* = 4.133, SD = 0.499, *p* < 0.001) and those with a doctoral degree (*M* = 3.390, SD = 0.434).
(4) Professional title: An independent samples *t*-test indicated a significant difference based on gender. Levene’s test indicated unequal variances *F*(3, 595) = 12.717, *p* < 0.001, η^2^ = 0.060. *Post hoc* comparisons revealed that teachers with a Senior title (*M* = 4.517, SD = 0.503) demonstrated significantly higher digital literacy compared to those with Junior (*M* = 4.231, SD = 0.428, *p* < 0.001), Intermediate (*M* = 3.931, SD = 0.624), no title (*M* = 4.031, SD = 0.523), Interestingly, the relationship between rank and competency was not strictly linear; teachers with junior titles scored significantly higher than both intermediate title holders and those with no title.

##### 4.1.2.2 Analysis of factors influencing preschool teachers’ digital competence

**FIGURE 2:**
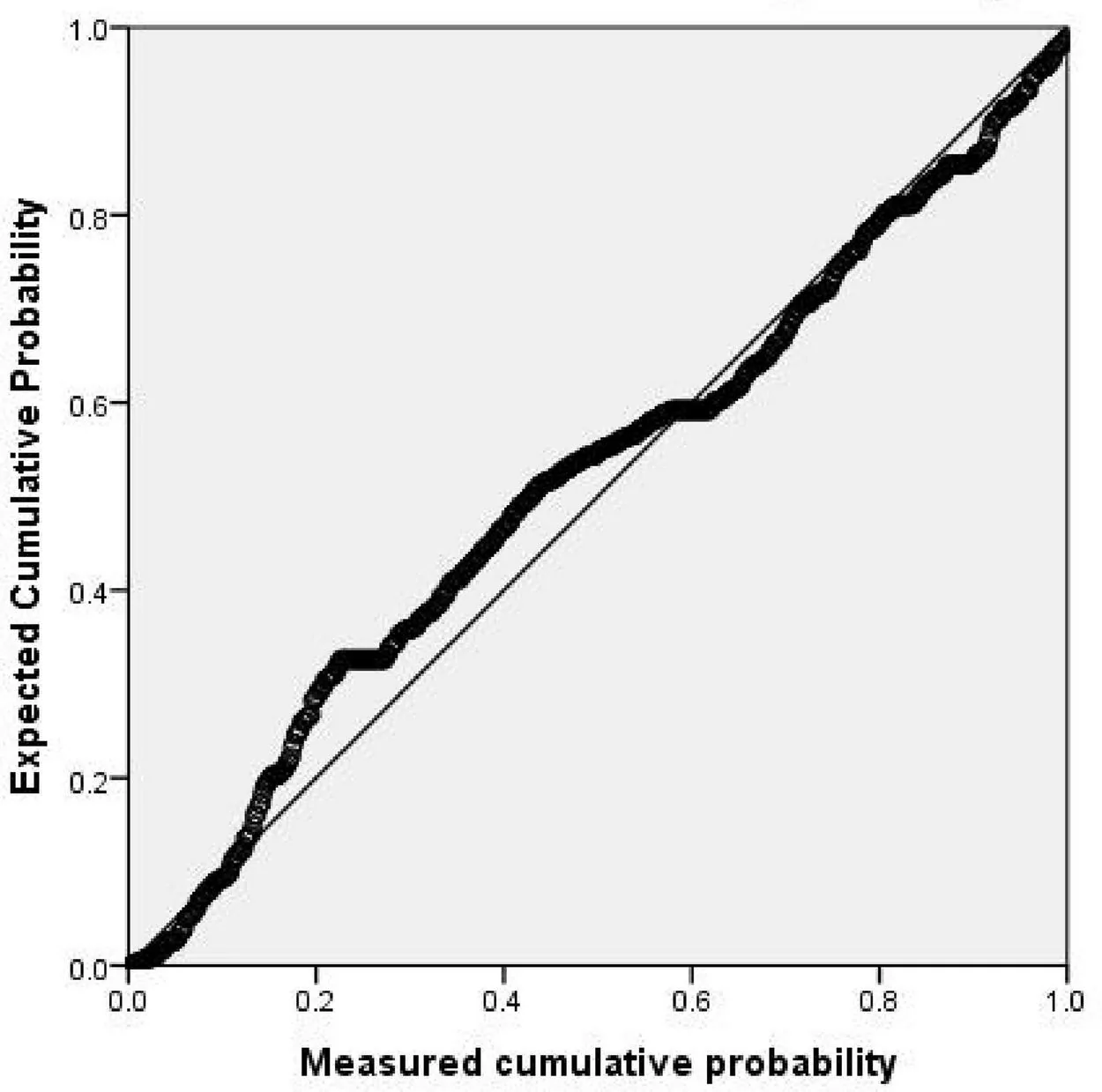
Normal P-P plot of regression standardized residuals. Dependent variable: mean score of the overall digital literacy dimension.

**FIGURE 3:**
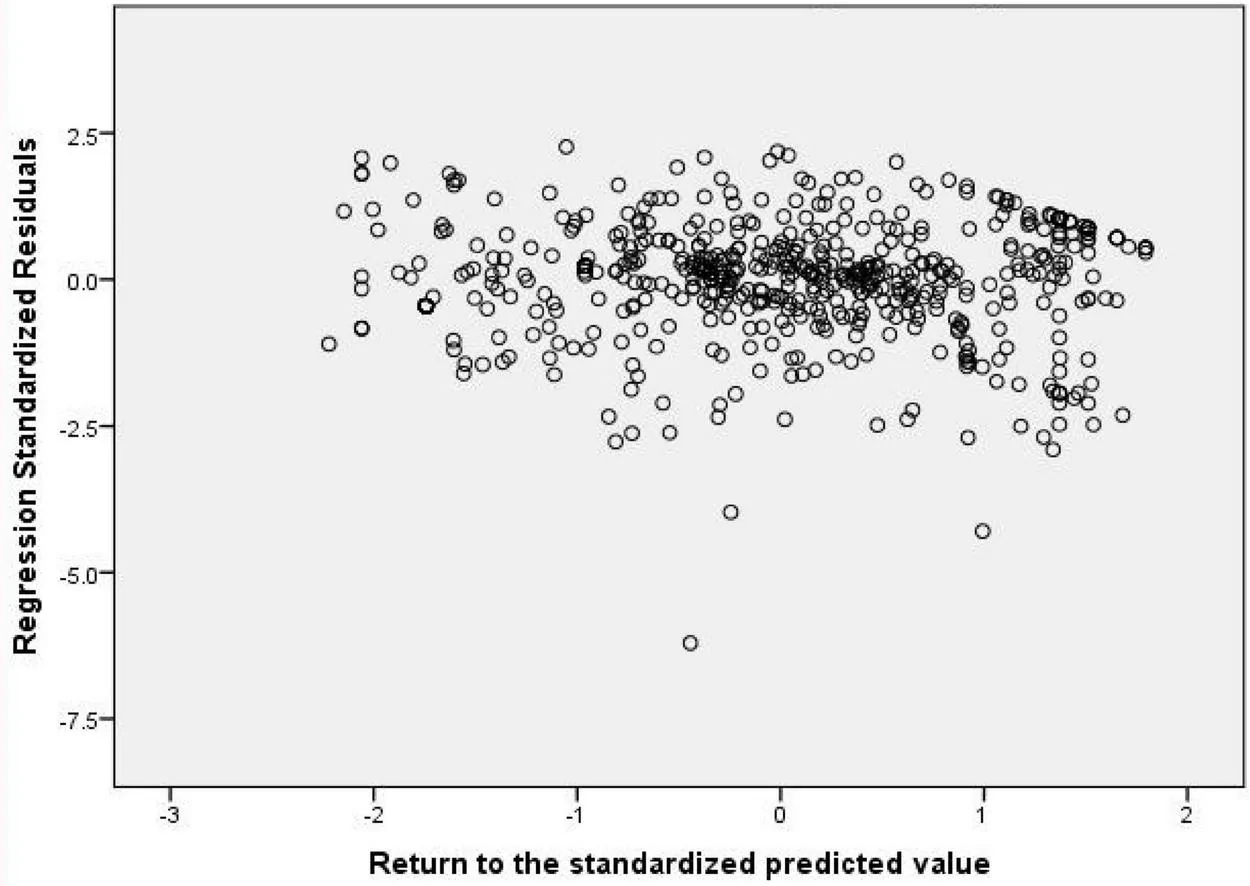
Scatter diagram. Dependent variable: mean score of the overall digital literacy dimension.

A one-way analysis of variance (ANOVA) was performed using the scores of preschool teachers’ overall digital competence and its sub-dimensions. The overall dimension served as the dependent variable, while the potential influencing factors were treated as independent variables (details information can be found in Tables 6, 7 and Figures 2, 3).

**TABLE 6:** Difference analysis of different predictive variables on the impact of preschool teachers’ digital literacy.

Coefficient^a^								
				Unstandardized coefficient		Standardized coefficient	*t*	*P*
Model	Variables	N	R^2^	B	S.E.	β		
1	Educational Level–>X	0.263^a^	0.069	−0.282	0.042	−0.263	−6.665	<0.001***
2	Educational level	–	–	−0.3	0.042	−0.28	−7.156	<0.001***
	Teaching years	0.310^b^	0.096	0.108	0.026	0.165	4.226	0.843
3	Educational Level	–	–	−0.237	0.038	−0.221	−6.268	<0.001***
	Teaching years	–	–	0.077	0.023	0.118	3.358	0.001**
	Motivation attitude	0.528^c^	0.279	0.365	0.03	0.433	12.263	<0.001***
4	Educational level	–	–	−0.213	0.031	−0.199	−6.95	< .001***
	Teaching years	–	–	0.052	0.019	0.08	2.806	.005**
	Motivational attitude	–	–	0.059	0.03	0.071	2.011	.045*
	Efficacy	0.727^d^	0.529	0.525	0.03	0.623	17.781	<0.001***
5	Educational level	–	–	−0.174	0.03	−0.162	−5.843	<0.001***
	Teaching years	–	–	0.057	0.018	0.087	3.178	0.002**
	Motivational attitude	–	–	−0.015	0.03	−0.018	−0.512	0.609
	Efficacy	–	–	0.351	0.036	0.417	9.653	<0.001***
	Proactive personality trait	0.755^e^	0.570	0.3	0.04	0.341	7.543	<0.001***
6	Educational level	–	–	−0.173	0.029	−0.161	−5.854	<0.001***
	Teaching years	–	–	0.055	0.018	0.084	3.108	0.002**
	Motivational attitude	–	–	−0.02	0.03	−0.024	−0.676	0.499
	Efficacy	–	–	0.314	0.038	0.373	8.255	<0.001***
	Proactive personality trait	–	–	0.281	0.04	0.32	7.034	<0.001***
	Artificial intelligence anthropomorphism	0.760^f^	0.578	0.096	0.031	0.108	3.152	0.002**
7	Educational level	–	–	−0.16	0.029	−0.15	−5.493	<0.001***
	Teaching years	–	–	0.056	0.017	0.086	3.234	0.001**
	Motivational attitude	–	–	−0.035	0.029	−0.041	−1.174	0.205
	Efficacy	–	–	0.303	0.038	0.36	8.071	<0.001***
	Proactive personality trait	–	–	0.236	0.041	0.268	5.811	<0.001***
	Artificial intelligence anthropomorphism	–	–	0.028	0.034	0.031	0.816	<0.001***
	Organizational support	0.769^g^	0.587	0.154	0.034	0.181	4.482	0.679

*N* = 450. *B*, unstandardized regression coefficient; S.E., standard error; β, standardized regression coefficient.

*P = 0.05,

**p = 0.01,

***p = 0.001.

^a^Predictor variable: (constant), 3. What is your educational background?

^b^Predictor variables: (constant), 3. What is your educational background? 8. How many years of teaching experience do you have?

^c^Predictor variables: (constant), 3. What is your educational background? 8. How many years of teaching experience do you have? Motivation and attitude.

^d^Predictor variables: (constant), 3. What is your educational background? 8. How many years of teaching experience do you have? Motivation attitude, efficacy.

^e^Predictor variables: (constant), 3. What is your educational background? 8. How many years of teaching experience do you have? Motivation attitude, efficacy, proactive personality traits.

^f^Predictor variables: (constant), 3. What is your educational background? 8. How many years of teaching experience do you have? Motivation attitude, efficacy, proactive personality traits, AI anthropomorphism.

^g^Predictor variables: (constant), 3. What is your educational background? 8. How many years of teaching experience do you have? Motivation attitude, efficacy, proactive personality traits, AI anthropomorphism, organizational support.

**TABLE 7:** Regression model analysis of influencing factors on digital competence of preschool teachers.

Model summary									
Model	R	R^2^	Adjusted R^2^	Error in standard estimation	Change statistics				
					R^2^change amount	F change amount	Degrees of freedom 1	Degrees of freedom 2	Significant F change
1	0.263^a^	0.069	0.068	0.502	0.069	44.418	1	597	0.000
2	0.310^b^	0.096	0.093	0.495	0.027	17.859	1	596	0.000
3	0.528^c^	0.279	0.275	0.443	0.182	150.374	1	595	0.000
4	0.727^d^	0.529	0.526	0.358	0.251	316.162	1	594	0.000
5	0.755^e^	0.570	0.567	0.342	0.041	56.904	1	593	0.000
6	0.760^f^	0.578	0.573	0.340	0.007	9.933	1	592	0.002
7	0.769^g^	0.591	0.587	0.334	0.014	20.090	1	591	0.000

^a^Predictor variable: (constant), 3. What is your educational background?

^b^Predictor variables: (constant), 3. What is your educational background? 8. How many years of teaching experience do you have?

^c^Predictor variables: (constant), 3. What is your educational background? 8. How many years of teaching experience do you have? Motivation and attitude.

^d^Predictor variables: (constant), 3. What is your educational background? 8. How many years of teaching experience do you have? Motivation attitude, efficacy.

^e^Predictor variables: (constant), 3. What is your educational background? 8. How many years of teaching experience do you have? Motivation attitude, efficacy, proactive personality traits.

^f^Predictor variables: (constant), 3. What is your educational background? 8. How many years of teaching experience do you have? Motivation attitude, efficacy, proactive personality traits, AI anthropomorphism.

^g^Predictor variables: (constant), 3. What is your educational background? 8. How many years of teaching experience do you have? Motivation attitude, efficacy, proactive personality traits, AI anthropomorphism, organizational support.

(1) Preliminary analysis: Prior to the regression analysis, assumptions were tested. The Normal P-P Plot of regression standardized residuals indicated that the residuals were approximately normally distributed. Furthermore, the scatterplot of standardized predicted values against standardized residuals showed a random dispersion of points, confirming homoscedasticity and linearity. No multicollinearity issues were detected (VIF = 1.031–3.082, VIF < 5).
(2) Hierarchical regression analysis: A hierarchical regression analysis was conducted to examine the predictive effects of demographic variables and psychological factors on preschool teachers’ digital literacy. The results are summarized in Table 6.

###### 4.1.2.2.1 Step 1: demographic variables (Models 1–2)

In Model 1, educational level was entered as a control variable and accounted for 6.9% of the variance in digital literacy (R^2^ = 0.069, *F* = 45.03, *p* = 0.001). Educational level significantly negatively predicted digital literacy (β = 0.263, *p* = 0.001). In Model 2, teaching years were added, increasing the explained variance by 24.2% (ΔR^2^ = 0.242). Both educational level (β = 0.280, *p* = 0.001) and teaching years (β = 0.165, *p* = 0.001) were significant predictors.

###### 4.1.2.2.2 Step 2: psychological factors (Models 3–7)

Subsequent models introduced motivational and personality variables. - Model 3 and 4: Motivational attitude initially showed a significant positive effect (β = 0.433, *p* = 0.001). However, upon adding self-efficacy in Model 4, the influence of motivational attitude weakened considerably. - Model 5 (Adding Proactive Personality): Self-efficacy emerged as a strong predictor (β = 0.417, *p* = 0.001), while motivational attitude became non-significant (β = 0.018, *p* = 0.609), suggesting its effect may be mediated or suppressed by self-efficacy. Proactive personality also contributed significantly *(*β = 0.341, *p* = 0.001). The model explained 52.9% of the variance. - Model 6 (Adding AI Anthropomorphism): AI anthropomorphism was a significant positive predictor (β = 0.108, *p* = 0.01). - Model 7 (Full Model - Adding Organizational Support): In the final model, organizational support was added. The full model explained 58.7% of the variance in digital literacy (R^2^ = 0.587, *F* = 86.68, *p* = 0.001).

###### 4.1.2.2.3 Final predictors (Model 7)

In the final model, organizational support was added. The full model explained 58.7% of the variance in digital literacy (R^2^ = 0.587, *F* = 86.68, *p* < 0.001). Four variables remained significant predictors:

(1). Self-efficacy: The strongest predictor (β = 0.360, *p* = 0.001, VIF = 2.870).
(2). Proactive personality: A moderate positive predictor (β = 0.268, *p* = 0.001, VIF = 3.082).
(3). Organizational support: A significant positive predictor (β = 0.181, *p* = 0.001, VIF = 2.349).
(4). Educational level: Remained a significant negative predictor (β = 0.150, *p* = 0.001, VIF = 1.074).

Teaching years showed a small but significant positive effect (β = 0.086, *p* = 0.001, VIF = 1.031).

Notably, motivational attitude (β = 0.041, *p* = 0.241, VIF = 1.769) and AI Anthropomorphism (β = 0.031, *p* = 0.415, VIF = 2.055) were no longer significant in the presence of the other strong predictors.

#### 4.1.3 The second research question

The study’s second question is, “What is the level of preschool teachers’ digital competence in China according to across five dimensions? To answer this question, We analyzed of 599 preschool teachers’ questionnaire, through correlation analysis, that their levels of digital competence generally fell within the upper-middle range, consistent with normal distribution characteristics. The overall score for preschool teachers’ digital competence is (4.102 ± 0.520), with scores across all dimensions exceeding three points. This finding indicates that while kindergarten teachers possess a foundational level of digital literacy, there remains significant potential for enhancement.

This study conducted a correlation analysis of five dimensions: digital literacy foundation, educational ability, learning ability, leadership, and management ability, based on four background variables: gender, education level, professional title, and teaching experience. Table 4 indicates that the aforementioned background factors are significantly correlated with both overall digital literacy and its individual dimensions. Specifically, digital competence among preschool teachers shows a significant negative correlation with education level (*r* = −0.263, *p* < 0.001). In contrast, it exhibits significant positive correlations with digital Competence foundation (*r* = 0.855, *p* < 0.001), Teaching ability (*r* = 0.929, *p* < 0.001), learning ability (*r* = 0.937, *p* < 0.001), leadership (*r* = 0.907, *p* < 0.001), and gender (*r* = 0.106, *p* < 0.01). Although the correlation with professional title reaches statistical significance (*r* = 0.078, *p* < 0.05), the effect size is relatively weak (details information can be found in Table 8).

**TABLE 8:** Correlation analysis of digital competence dimensions and background information in preschool teachers.

Variables	M ± SD	1. Sex	2. Educational level	3. Professional title	4. Teaching years	5. Digital competence– BS	6. Digital competence – teaching	7. Digital competence– TATL	8. Digital competence– Leadership	9. The average score for TOADOL
1	3.981 ± 0.055 (female)	1	–	–	–	–	–	–	–	–
	4.127 ± 0.023 (male)									
2	4.133 ± 0.499	−0.501***	1	–	–	–	–	–	–	–
3	4.03 ± 0.523	−0.358***	0.512***	1	–	–	–	–	–	–
4	4.054 ± 0.031	−0.08	0.103*	0.334***	1	–	–	–	–	–
5	4.138 ± 0.567	0.221**	−0.359***	−0.041	0.167**	1	–	–	–	–
6	4.186 ± 0.530	0.112**	−0.233**	0.079	0.155**	0.766***	1	–	–	–
7	4.062 ± 0.565	0.024	−0.167**	0.120**	0.119**	0.724***	0.824***	1	–	–
8	4.021 ± 0.631	0.035	−0.200**	0.120**	0.064	0.625***	0.795***	0.849***	1	–
9	4.102 ± 0.520	.106**	−0.263**	0.078	0.137**	0.855***	0.929***	0.937***	0.907***	1

BS, basics kills; TATL, the ability to learn; TOADODL, the overall dimension of digital literacy;

*p < 0.05;

**p < 0.01;

***p < 0.001.

### 4.2 The qualitative section

This section was contributed to answer the third question, “What are the perceptions of preschool teachers in China toward using digital tools in the educational environment?”

#### 4.2.1 Perceived benefits of using digital tools in preschool education

##### 4.2.1.1 Enhancing pedagogy through interactivity and visualization

Our research indicates that interactive and visual teaching approaches facilitate the development of localized curriculum designs in kindergartens. Such kindergarten-specific curricula are closely aligned with children’s daily lives, and their thematic activities are designed to be easily comprehensible for young learners. When thematic activities are highly abstract, teachers’ digital literacy enables them to visualize activity scenarios and make content more relatable to everyday life; by creating interactive or real-world contexts, this approach directly aids children in understanding the activity themes.

e.g., Teachers reported using multimedia resources to create contextualized learning scenarios that would be helpful for children’s understanding. As one principal (P6) noted, “teachers utilize images, sounds, and videos to create contextualized scenarios, which helps in visualizing complex ideas for young children.”

This perspective aligns with that of a teacher (T2): “When helping young children understand day and night, I use visual picture books and children’s science animations. When these resources are unavailable online, I also employ GenAI tools like Doubao to generate supplementary material such as images, short videos, and story scripts. Through visual aids, children can easily grasp the concepts of day and night.”

This approach is further supported by institutional partnerships: for instance, a principal (P1) said, “Lenovo Group developed an interactive software application specifically for our kindergarten and proactively provided training for early childhood educators. Using this tool, teachers have created numerous engaging thematic activities that broaden children’s horizons and enhance the learning atmosphere during sessions. Children also thoroughly enjoy these interactive learning experiences, which align perfectly with their natural inclination toward gamified and visually intuitive learning methods.”

This indicates a clear perception among educators that digital tools, when applied effectively, can bridge the gap between abstract curriculum goals and children’s concrete understanding.

##### 4.2.1.2 Management efficiency and professional willingness of preschool teachers

In our study, we found that teachers have positive attitudes toward digital tools. For example, the teacher (T1) who is a teacher from a kindergarten in the southern part of China, shared that, I would use it when I am confident, I can use the tool proficiently and I have received relevant training, Besides, demonstrably improves teaching outcomes, I am more likely to adopt it.

Similarly, another teacher (T6) in the eastern part of China, who also pointed out that, “If I become proficient with this digital tool, I will be more confident in applying it to the early childhood thematic activities I design. For instance, by utilizing the school’s newly introduced digital devices, I can create more engaging and immersive scenarios for interaction with young children. Taking our body-themed activities as an example, I could develop a visual animated adventure that allows children to explore body parts interactively and engage in virtual dialogues about how to better protect their bodies.”

This sentiment was echoed by Principal (P2), “who observed that when teachers witness digital resources successfully enhancing children’s interaction and understanding, their own willingness to adopt and integrate these tools grows significantly. Especially when kindergartens provide specialized training on such equipment, teachers’ willingness to use it significantly increases.”

On the other hand, our research found that many early childhood educators believe digital tools can help alleviate their workload and improve work efficiency. The teacher (T9) in the eastern part of China, who also pointed out that, “For example, with parental consent, our school has implemented a smart monitoring system. Each child is equipped with a smart wristband designed primarily to monitor and record their body temperature, activity participation rate, sleep patterns, respiratory parameters, heart rate, and detect any abnormalities. This data-driven approach reduces teachers’ workload and facilitates more effective home-school communication.”

This demonstrates that teachers are willing to utilize digital tools when they believe these tools can enhance their work efficiency and reduce daily workload burdens. Particularly after receiving training, when teachers develop a sense of confidence (“I can do it”), their frequency of using digital tools increases significantly.

#### 4.2.2 Perceived challenges of using digital tools in preschool education

##### 4.2.2.1 Insufficient professional development for teachers to apply functions through the interactive design

Our research indicates that proactive organizational support and comprehensive professional training can enhance their ability to utilize digital tools. However, without systematic training, their willingness to employ digital tools in designing thematic activities remains low.

For example, a teacher (T3) pointed out: “When assigning tasks to teachers, our school instructed us to proactively utilize digital tools for digital scenarios—such as creating AI-powered interactive videos for children using tools, such as Doubao and Jimeng. However, due to a lack of clear guidelines and detailed instructions, we lacked the initiative to fully explore these tools.”

Another teacher (T8) added: “I only actively learn these digital tools when facing pressure for professional title advancement or when delivering open-class demonstrations; otherwise, I lack the motivation to explore them proactively. This is also due to a lack of comprehensive training and insufficient proficiency in using these emerging digital tools for activity design.”

This indicates that preschool teachers’ willingness to use digital tools depends on whether they receive comprehensive and ongoing trainin.

##### 4.2.2.2 Difficulties caused by inconsistent digital systems across providers

During our interviews, we observed a common phenomenon: kindergartens select digital tool suppliers through phased bidding processes. Different suppliers offer digital tools with varying design concepts and usage methods, often requiring comprehensive support services—including after-sales assistance—to train teachers and address operational issues. Upon service termination and supplier replacement, teachers must relearn how to use the new digital tools and systems.

The teacher (T5) notes that “Since I joined the kindergarten, I have switched suppliers of digital tools three times. Only after receiving comprehensive training and becoming proficient in using these tools can we proactively employ digital tools for activity design. However, once the service cycle concludes, we are often forced to undergo training on new digital tools and new service providers. The upside is that this approach better aligns with evolving technological trends; the downside is that during training sessions for new tools, staff often struggle with adaptation due to frequent system changes.”

##### 4.2.2.3 Unequal access across kindergartens to digital tools

We found that Digital tools and resources in kindergartens are distributed unevenly. Governments and businesses prioritize funding or support for kindergartens with higher ratings. Due to receiving more funding, these institutions update their digital tools and equipment faster than other kindergartens.

A kindergarten principal (P5) pointed out, “Our kindergarten is located in the central region of China and ranks highly locally; our digital tools are also advanced in local. However, we can clearly perceive a gap compared to kindergartens in the eastern regions. During a previous visit to a kindergarten in Shanghai, we observed that their smart equipment was highly comprehensive, the visual presentation during open classes was excellent, and children responded very positively to activities. We felt truly envious.”

Similarly, within kindergartens, there is also an uneven distribution of resources, with key teachers receiving more training and possessing qualifications in core digital technologies.

A teacher (T4) point that resource allocation is often unequal.

“New teachers typically handle fundamental tasks such as student information processing, basic digital operations, video recording, and editing. They often lack the ability to apply or adapt digital technologies in designing activity-based learning programs for young children.”

An introduction by a key kindergarten teacher (T7): “Each of our external visits and training programs is subject to budget and quota constraints. As a key teacher, you have greater opportunities for external exchanges and training, as well as more access to digital technology. The most distinctive feature of key teachers is their clear career advancement pathways; this professional progression pressure. In turn, places higher demands on their digital teaching capabilities.”

In response, the principal (P4) expressed agreement.

“This creates a stratified environment where digital competence is not developed uniformly but is concentrated among a select group, leaving others, particularly young teachers without formal titles, with fewer opportunities to grow.”

A principal located in central China (P3) added, “Many early childhood educators face pressure to advance their professional titles. Senior teachers often prefer participating in training programs and exchange visits on behalf of their institutions, making them more likely to receive advanced digital technology training. In contrast, intermediate-level teachers only proactively enhance their digital competence and improve classroom effectiveness when under promotion pressure or during public demonstration classes. Additionally, I have observed that male teachers primarily demonstrate proficiency in ICT skills, while female teachers excel at designing more detailed interactive scenarios based on observations of young children.”

These findings indicates an uneven distribution of resources such as digital tools in kindergartens, which hinders the improvement of early childhood educators’ digital competence.

## 5 Discussion

Digital competence is essential for kindergarten teachers. This study employed a mixed-methods approach to examine how digital competencies manifest among preschool educators and to identify their principal influencing factors. We propose a comprehensive theoretical framework comprising five dimensions—digital foundational competence, digital teaching competence, digital learning competence, digital leadership, and digital management competence.

The results indicate that improving preschool teachers’ digital competence requires a dual approach: internally enhancing self-efficacy, motivation, and proactive traits, and externally promoting anthropomorphic AI usage, upgrading equipment, and providing organizational support.

### 5.1 The level of digital competence among preschool teachers

Our study found that preschool teachers’ overall digital competence was relatively high, especially in digital management capability (4.323 ± 0.594), suggesting a link to kindergartens’ digitalization level. This aligns with prior research: a Chinese survey indicated that most kindergarten principals view higher digital management as supporting informatization, recording children’s activity data, assisting teachers’ digital practices, and improving digital literacy (Wang and Fan, 2024).

The second dimension, “digital teaching cultivation” (4.186 ± 0.530), indicates that preschool teachers consider themselves capable of using digital technologies to organize children to participate in activities. This aligns with Yang and Hong (2022), who argue that teachers’ digital skills mainly help children perceive the real world and meet daily needs. Empirical evidence confirms that integrating digital technologies into curricula enhances learning outcomes, teaching effectiveness, and teacher–child interaction during thematic activities (Mertala, 2019).

The next dimension, “digital foundational competence” (4.138 ± 0.568), shows that preschool teachers possess basic digital skills, defined as the ability to use fundamental ICT to support education. Some scholars also highlighted that kindergarten teachers can use tools like whiteboards and slides to interact with children and enrich their perceptual experiences (Yu and Li, 2023).

Preschool teachers performed well in “digital learning capacity” (including motivational attitudes and efficacy), though slightly lower than other dimensions. This suggests they need to strengthen intrinsic motivation and self-efficacy in using digital technologies. These results align with prior research showing that teachers’ self-efficacy and confidence in applying ICT directly influence their proficiency (Morris et al., 2017; Weigold and Weigold, 2021; Gözüm et al., 2023). Perceived self-efficacy is as important as actual competence because it shapes teachers’ knowledge, skill levels, and capacity to integrate ICT. Conversely, teachers’ ICT self-efficacy also affects the quality of early childhood education (Gözüm et al., 2023). Furthermore, digital learning capacity is linked to motivation to adopt ICT, especially when teachers view their skills as effective solutions to teaching challenges and tools to increase engagement (Medina-García et al., 2021) Together, these findings emphasize the need for ongoing professional development in acquiring digital technologies, refining skills, and applying them in practice among preschool educators.

Our study found that preschool teachers performed strongly in “digital leadership” (policy and organizational support) but scored lowest among the five competency dimensions. This indicates persistent difficulties in information management and diagnostic assessment. Meanwhile, China’s recent policies: *Smart Education White Paper Teacher-Generated AI Guidelines* (2025), emphasize the need for preschool teachers to align with national digital competence policies, necessitating digital technology training and the use of digital tools in daily childcare and educational management to strengthen their digital leadership. To create more effective educational ecosystems, funding for professional development must be increased, and policies that encourage collaborative teacher communities must be implemented.

From the perspective of the overall framework: the strong positive correlations among the five dimensions (r = 0.85) validate the structural integrity of our framework. From a TPACK perspective, Digital Foundation (analogous to Technological Knowledge, TK) serves as the prerequisite for Digital Nurturing (analogous to Technological Pedagogical Content Knowledge, TPACK) (Herman et al., 2025). The scores indicate that teachers are relatively confident in Digital Foundation (technical operations) and Digital Nurturing (pedagogical application), which represent the “tip of the iceberg model.” However, the lower relative scores in Digital Leadership This indicates that although the teacher possesses A1 (Newcomer) and A2 (Explorer) skills, there remains a significant gap compared to the C1 (Leader) and C2 (Pioneer) levels (Redecker, 2017).

### 5.2 The difference of digital competence among preschool teachers

Our research reveals disparities in digital competence among preschool teachers. Overall, the general level is relatively high. Across five dimensions, digital management is the strongest, followed by foundational skills, teaching competence, learning skills, and leadership. Background factors such as gender, educational attainment, professional title, and teaching experience are significantly correlated with digital competence (*p* < 0.001). Educational level (η^2^ 0.111) is the most influential factor, followed by professional title, teaching experience, and gender.

Our study revealed that the proportion was highest among Master’s degree, followed by Bachelor’s degree or below and Doctoral degree. This result contradicts prior work showing that teachers with Master’s degree demonstrate stronger digital competence (Kozak et al., 2024; Li and Wang, 2025). Some studies suggests that digital competence in kindergarten practice primarily focuses on technical knowledge (TK) and the deep integration of technology with specific teaching contexts (TPACK) (Masoumi, 2021; Valdez and Mendoza, 2024; Herman et al., 2025).

The second important factor is teaching experience (η^2^ 0.021). Our study found that teachers with more than 11 years of experience exhibited the highest digital literacy (*F* = 4.286, *p* < 0.001). This demonstrates that as teaching experience accumulates, teachers’ ability to integrate technology with instruction (TPACK) continues to improve (Herman et al., 2025). Interestingly, teachers in the 8–10 year range showed significantly higher competence than novices (*p* < 0.05). This finding suggesting that professional accumulation plays a key role after the initial survival phase of teaching. Consistent with Mertala’s (2019) call for targeted training for early-career teachers, our findings highlight a clear experience gap. While novices often rely on basic ICT for instruction, experienced educators leverage advanced tools—such as facial recognition, interactive picture books, and digital portfolios—to deepen learning and strengthen home–school communication. Finally, it helps teachers achieve C1 (Leader) and C2 (Pioneer) levels (Redecker, 2017).

We found that teachers with senior professional titles demonstrated the highest level of digital competence, next comes the Junior title (3 < 1 < 2 < 4^**^). Senior (4.517) > Junior (4.231) > No title (4.031) > Intermediate (3.931). However, it is noteworthy that intermediate-level teachers exhibited a significant “low point,” with their scores markedly lower than those of teachers without professional titles or holding junior titles. This may suggests that intermediate-level teachers need to carry out a “knowledge reorganization” of subject knowledge (CK) and pedagogical knowledge (PK) in accordance with the TPACK framework (Herman et al., 2025). The second most common category is “No title” likely because novice teachers’ teaching methodologies are not yet well-established, making it easier for them to directly incorporate technical skills into their instruction. This pattern contrasts somewhat with the conclusions of Chen and Min’s (2024), who reported that teachers with junior titles exhibited significantly higher digital awareness than those with intermediate or senior titles, with no significant differences in other dimensions.

Finally, our study found that female preschool teachers demonstrated higher levels of digital competence, consistent with Aguaded et al. (2015) but at odds with the views of some scholars (Roig. Women outperformed men in digital competences related to instructional organization, whereas men reported higher perceived value of digital devices and higher self-assessed competence (Gabarda Méndez et al., 2023)].

During the interview, a principal (P3) also highlighted the presence of gender-based division of responsibilities among frontline teachers. Male preschool teachers more frequently hold teaching or administrative roles and primarily serve auxiliary functions in instructional activities. Female teachers have developed more effective digital visualization tools tailored to the actual needs of young children. In contrast, some teachers noted that although male educators may be more attuned to digital tools and curriculum design principles, their effectiveness in designing and implementing transgender education programs may be inferior to that of female teachers.

### 5.3 The key influence factors of digital competence among preschool teachers

Our findings indicate that the following factors influence preschool teachers’ digital competence, ordered from most to least influential: self-efficacy, motivation, proactive personality traits, organizational support, and AI anthropomorphism. These findings align with the underwater characteristics described in the iceberg model, indicating that early childhood educators’ learning competence is influenced by these factors (Salleh et al., 2015).

Self-efficacy showed the strongest predictive power (β = 0.525, *p* < 0.001). Bandura’s social cognitive theory suggests that high self-efficacy enables teachers to achieve quality teaching outcomes (Bandura, 1997). Relevant ICT studies corroborate this finding. A study found that all measurement items were significantly correlated with and discriminated between levels of self-efficacy and ICT proficiency(TPACK) (Herman et al., 2025); teachers with higher proficiency were more likely to use digital resources to create interactive learning environments study found that teachers with strong self-efficacy were better at designing challenging curricula, managing pupil behavior effectively, and promoting pupils’ active participation in learning activities, thereby fostering higher-quality learning environments (Tsouloupas et al., 2010).

Our study identified motivation as the second-strongest predictor and found a strong positive correlation between motivation and digital competence. This indicates that preschool teachers with higher motivation typically demonstrate greater digital competence. This finding is consistent with a scholar, who showed that in gamified curricula, external rewards with collectible or high motivational value—such as tokens, coins, and group collaboration—enhance users’ digital competence (Fabre-Mitjans et al., 2025). These findings indicate that both extrinsic motivations (e.g., career advancement) and intrinsic motivations (e.g., self-efficacy) encourage preschool teachers to proactively improve their digital competencies. Proactive personality traits significantly impact preschool teachers’ digital competence; individuals with stronger proactive personalities are more likely to embrace digital technologies and demonstrate higher digital competence. From the perspective of the TPACK framework, proactive teachers not only possess technical knowledge (TK), but also strive to integrate it with pedagogical knowledge (PK) and subject-specific knowledge (CK) (Masoumi, 2021; Herman et al., 2025). Proactive personality, introduced by Bateman and Crant (1993), refers to a stable disposition that enables individuals to overcome environmental constraints and reshape their surroundings through enhanced environmental-intervention capabilities, self-efficacy, and a change-adaptive attitude. Studies suggest that proactive teachers are more likely to explore teaching strategies and adopt positive attitudes toward diverse educational approaches (Cui and Liu, 2025). And effectively utilize digital technology for educational purposes, thereby exhibiting high digital competence (Papavlasopoulou et al., 2024). These findings underscore the importance of active behavior in enhancing individuals’ sense of competence (Wu et al., 2018).

Our findings indicate that organizational support also affects preschool teachers’ digital competence. Skantz-Åberg et al. (2022) emphasize that such support should be available to all teachers, regardless of training background, and should include access to basic digital infrastructure and training. Research has confirmed that organizational support serves as a critical driving factor. This is similar to the iceberg model, where the tissue corresponds to the “water surface” layer of the barrier, the environmental context that either facilitates or impedes behavioral outcomes (Ahrens, 2015; Salleh et al., 2015).

We found that kindergarten teachers receiving strong organizational support exhibit higher digital competence; institutional training and managerial recognition markedly increase their motivation to develop these skills. This aligns with Pinto-Santos et al. (2022), who found that teachers with robust organizational support more frequently use active teaching methods. Relevant ICT research corroborates these results: studies have shown that ICT training for preschool teachers raises their enthusiasm for technology use and provides them with the skills needed to support children’s learning. Dardanou et al. (2023) highlight the importance of structured programmes, such as pre-service training, in enabling teachers to master ICT teaching methods and competencies—a perspective supported by the ECTE project. The study reports that the ECTE programme enhanced digital competence by integrating knowledge of digital technologies and providing pre-service early childhood teachers with opportunities to work with children using technology during internships.

This study found that AI anthropomorphism also enhanced preschool teachers’ digital competence. Teachers proficient in using AI tools exhibited higher levels of digital competence: they not only used AI platforms such as Youku Tang and Qianwen for lesson preparation but also employed anthropomorphic AI interactions to design classroom scenarios and manage lessons effectively. AI anthropomorphism enabled adaptive feedback, allowing AI tools to think, interact, and provide responses efficiently (Choi and Lee, 2018). This indicates that preschool teachers are actively embracing AI anthropomorphism to improve their digital competence. This finding aligns with the development trends of DigCompEdu (Redecker, 2017).

## 6 Implications

– Theoretically, our study identified several core influencing factors: self-efficacy, motivation, proactive personality traits, organizational support, and AI anthropomorphism. These findings corroborate the “iceberg theory,” showing that the latent personal traits posited by that theory substantially affect teachers’ digital competence and emphasize the importance of individual factors for digital capability. Organizational support must also be prioritized. Relevant ICT studies indicate that such support plays a crucial role in enhancing the digital competence of preschool teachers. This aligns with the TAPCK perspective within our thematic framework. By means such as gamified instruction and contextual design, teachers were expected to apply intelligent technologies more effectively in specific classroom scenarios while attending to ethical considerations. This suggests that China’s education authorities have been attempting to strengthen organizational support for preschool teachers’ digital competence through policy measures.

In practice, our findings indicate variation in preschool teachers’ digital competence. Teachers demonstrate strong digital abilities across most dimensions but are weak in “Digital Learning Skills” and “Digital Management Skills.” This pattern shows that preschool teachers are proficient in using basic digital devices for childcare and education and in handling student information, but they have deficiencies in self-efficacy, motivation, and organizational support. Consequently, priority should be given to addressing teachers’ professional development needs by strengthening institutional training to boost their confidence in using digital technologies and by fostering intrinsic motivation for digital adoption. Preschool teachers with differing educational backgrounds, professional titles, ages, years of teaching experience, and genders showed variation in their digital competence levels. This suggests that support should be tailored to individual differences among preschool educators. This aligns with the perspectives on cultivating C1 (expert-level) and C2 (excellence-level) professionals outlined in the DigComEdu framework we use as our theoretical reference. We identified several key influencing factors, indicating that individual-level interventions should be prioritized to enhance teachers’ self-efficacy, thereby strengthening intrinsic motivation and encouraging proactive engagement with digital technologies. At the same time, institutions must provide external support and meet necessary requirements to supply teachers with the tools and resources required for skills development.

## 7 Limitation

This study examined preschool teachers’ digital competence—its manifestations and key determinants. The findings enrich the literature on digital competence in this population and provide a theoretical basis and practical guidance for designing systems to cultivate and support teachers’ digital competence, contributions that have clear practical significance. Nonetheless, the study has the following limitations.

Firstly, the study relied on self-reports, and reported the common method bias. Future research should use experimental designs or situational testing to obtain more comprehensive data and strengthen the reliability of findings. Such work should also investigate the internal mechanisms and principal drivers of preschool teachers’ digital competence to identify underlying constraints and to clarify how school support relates to competence levels.

Secondly, this study was cross-sectional, so it could not reveal trends in teachers’ digital competence over time or the predictive effects of different predictors. Future research should employ longitudinal studies to map the developmental trajectory of teachers’ digital competence and to examine the dynamic predictive effects of various factors.

Thirdly, uneven sample distribution. Although multiple regions were covered, no quotas balanced regional sizes. Since researchers were in Anhui, sampling bias may exist. Future studies should use stratified national sampling to increase size and balance representation, improving universality.

Finally, the study did not examine intrinsic correlation mechanisms. Current research has focused mainly on core predictors, while the mechanisms linking these factors to numerical abilities (for example, mediation and moderation effects) remain untested. Future studies should employ structural equation modeling to investigate these underlying mechanisms.

## 8 Conclusion

This study examined the manifestations and key factors of preschool teachers’ digital competence. Using statistical methods, we diagnosed the state and determinants of their digital literacy, developed assessment dimensions and a questionnaire for measuring preschool teachers’ digital literacy, and provided a tool for evaluating their developmental level. We found higher digital competence among teachers with senior professional titles, female teachers, those with Master’s degree, and those with longer teaching experience. Major factors affecting preschool teachers’ digital competence include efficacy, proactive personality traits, extrinsic motivation, and organizational support. These findings indicate that there are differences in the digital competency levels among preschool teachers. We need to provide tailored support, take into account the individual characteristics of preschool teachers, and enhance their digital competence.

## Data Availability

The original contributions presented in this study are included in this article/Supplementary material, further inquiries can be directed to the corresponding author.

## Footnotes

1 These five dimensions are selected from the questionnaires that scholars have already developed. In the second section, the questions are adapted from the “Primary and Secondary School Teachers’ Digital Competence Questionnaire” (Hu, 2023) and the “Teacher Digital Competence Survey Questionnaire” (Zheng, 2019), with reference to the “Primary and Secondary School Teachers’ Digital Literacy Assessment Questionnaire” (2023) developed by the Ministry of Education’s Research Base for Educational Informatization.
